# Comparison of Stereotactic Body Radiotherapy and Surgery for Stage I Lung Cancer: A Multidisciplinary Cohort Study Utilizing Propensity Score Overlap Weighting and AI-Based CT Imaging Analysis

**DOI:** 10.3390/cancers17122015

**Published:** 2025-06-17

**Authors:** Eun Hye Lee, Young Joo Suh, Jong Won Park, Jisu Moon, Sangjoon Park, Chang Geol Lee, Hong In Yoon, Byung Jo Park, Jin Gu Lee, Dae Joon Kim, Seung Hyun Yong, Sang Hoon Lee, Chang Young Lee, Jaeho Cho, Eun Young Kim

**Affiliations:** 1 Division of Pulmonology, Allergy and Critical Care Medicine, Department of Internal Medicine, Yongin Severance Hospital, Yonsei University College of Medicine, Yongin 16995, Republic of Korea; hieunhye@yuhs.ac; 2 Department of Radiology, Research Institute of Radiological Science, Severance Hospital, Yonsei University College of Medicine, Seoul 03722, Republic of Korea; rongzu@yuhs.ac; 3 Department of Radiation Oncology, Yonsei Cancer Center, Heavy Ion Therapy Research Institute, Yonsei University College of Medicine, Seoul 03722, Republic of Korea; jongwon987@yuhs.ac (J.W.P.); depecher@yuhs.ac (S.P.); cglee1023@yuhs.ac (C.G.L.); yhi0225@yuhs.ac (H.I.Y.); 4 BIostatistics Collaboration Unit, Department of Biomedical Systems Informatics, Yonsei University College of Medicine, Seoul 03722, Republic of Korea; labbios@yuhs.ac; 5 Thoracic and Cardiovascular Surgery, Severance Hospital, Yonsei University College of Medicine, Seoul 03722, Republic of Korea; bzpark@yuhs.ac (B.J.P.); csjglee@yuhs.ac (J.G.L.); kdjcool@yuhs.ac (D.J.K.); 6 Division of Pulmonary and Critical Care Medicine, Department of Internal Medicine, Yonsei University College of Medicine, Seoul 03722, Republic of Korea; roneirire@yuhs.ac (S.H.Y.); cloud9@yuhs.ac (S.H.L.)

**Keywords:** stage I non-small cell lung cancer, stereotactic body radiotherapy, surgery, recurrence, survival, treatment outcome

## Abstract

**Background:** With rising life expectancy and widespread lung cancer screening, early-stage non-small cell lung cancer (NSCLC) incidence has increased. While surgery is the standard treatment for operable stage I NSCLC, many patients are ineligible due to age or comorbidities. Stereotactic body radiotherapy (SBRT) has achieved good primary tumor control rates and overall survival. This study compares the outcomes of SBRT and surgery for stage I NSCLC using propensity score overlap-weighted dataset. **Methods:** This retrospective study analyzed clinical stage I NSCLC patients treated at a tertiary hospital from 2012 to 2021. Baseline differences between SBRT and surgery groups were adjusted using overlap weighting. AI-based CT analysis (CT AI-CAD) assessed tumor characteristics, verified by radiologists. Primary outcomes were 5-year cumulative incidence of recurrence and overall survival, with subgroup analyses based on tumor features. **Results:** Of 1474 patients, 1258 underwent surgery, and 216 received SBRT. After overlap weighting, baseline characteristics were well balanced. The 5-year cumulative incidence of recurrence and OS showed no statistically significant differences between SBRT and surgery groups (recurrence: 16.2% vs. 16.1%; OS: 80.5% vs. 82.9%). Further AI-based CT subgroup analysis showed no significant differences in recurrence rates across tumor features. A solid tumor diameter associated with a significant increase in recurrence was identified as 16.6 mm for SBRT and 18.6 mm for surgery. **Conclusions:** After overlap weighting, SBRT and surgery showed no statistically significant differences in treatment outcomes in stage I NSCLC. These findings may help guide the timing and selection of safe and effective treatment approaches.

## Simple Summary

This study compares stereotactic body radiotherapy (SBRT) and surgery in patients with stage I non-small cell lung cancer (NSCLC), using a robust overlap-weighted propensity score approach to address disparities in baseline characteristics. By incorporating artificial intelligence (AI)-based Chest CT features through computer-aided detection (CAD), the study provides a novel analysis of radiological tumor characteristics. No statistically significant differences in outcomes were found between SBRT and surgery, even after stratifying by tumor diameter, lobar location, or pleural attachment. Conducted in South Korea, a country with advanced lung cancer screening practices, the study highlights the clinical value of SBRT, especially for patients with comorbidities or limited surgical options. These findings may support more inclusive and personalized treatment strategies for early-stage lung cancer.

## 1. Introduction

With the active implementation of lung cancer screening, the number of cases diagnosed at an early stage has been increasing. The US National Cancer Database and Surveillance Epidemiology End Results (SEER) program reported that stage I non-small cell lung cancer (NSCLC) cases among patients aged 55–80 rose from 27.8% in 2010 to 35.5% in 2018 [1]. In South Korea, according to the 2020 Health Insurance Review and Assessment Service (HIRA) report [2], 31.4% of NSCLC cases are now identified at stage I, a significant increase from the 22.4% in 2013 [3]. The stage shift toward stage I NSCLC has led to improved survival. As one-third of lung cancer patients are aged 70 years or older [2], this shift underscores the need for effective, curative strategies that ensure long-term survival while considering overall health. It also highlights the growing necessity of tailored, viable treatments for elderly patients and those with comorbidities.

Surgery is the standard treatment for operable early-stage lung cancer [4]. However, certain patients who are elderly or have comorbidities make surgery unsuitable. As a result, there is a growing need to optimize non-surgical treatment options for these patients. Stereotactic body radiotherapy (SBRT) has been recommended as a standard treatment in international guidelines for patients who are medically inoperable, have surgical risks, or decline surgery following thoracic surgical consultation for stage I NSCLC [5]. SBRT offers a non-invasive option with lower toxicity, albeit with the potential risk of radiation pneumonitis [6]. Selecting the most appropriate treatment for patients with early-stage NSCLC often requires careful consideration of multiple factors, including the patient’s overall health, tumor characteristics such as size and location, and individual preferences. As the number of patients undergoing SBRT continues to rise, questions remain regarding its comparative long-term efficacy and survival outcomes relative to surgery, highlighting the importance of further research to define its role in tumor control and overall survival. Given that SBRT has become the standard option for patients who are either unable to undergo surgery or decline it, this study was designed to leverage accumulated real-world evidence to provide robust support for its proposed role in evolving clinical practice.

This study aims to compare the treatment outcomes of SBRT and surgery in patients with stage I NSCLC. Propensity score overlap weighting was applied to address baseline differences between SBRT and surgery groups, adjusting for demographics and tumor characteristics.

## 2. Materials and Methods

### 2.1. Patients and Clinical Data

**Figure 1:**
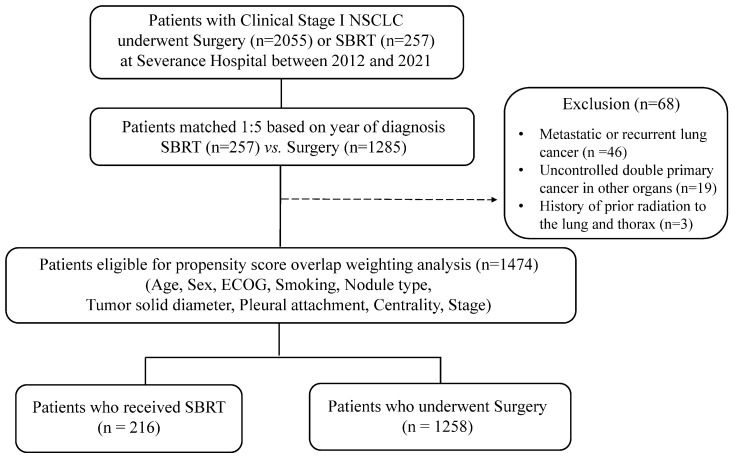
Study flowchart. Abbreviations: NSCLC, non-small cell lung cancer; SBRT, stereotactic body radiotherapy; ECOG, Eastern Cooperative Oncology Group.

Patients with clinical T1-2a (≤4 cm), N0M0, NSCLC who underwent SBRT or surgery at a tertiary referral hospital between 2012 and 2021 were analyzed retrospectively (Figure 1). Patients with metastatic or recurrent lung cancer, uncontrolled double primary cancer in other organs, or a history of prior radiation to the lung and thorax were excluded from the analysis. Lung cancer stage was assessed according to the 8th edition of TNM classification [7] and tumor response was assessed using the Response Evaluation Criteria in Solid Tumors (RECIST) version 1.1 [8]. Data on treatment outcomes were followed for 5 years until death or last follow-up for most patients; however, patients treated between 2020 and 2021 were followed for at least 3 years after their treatment date. This study was approved by the Institutional Review Board (IRB) of our hospital. The need for informed consent was waived by the IRB due to the retrospective nature of the study.

### 2.2. Treatment

Surgical resection was performed under general anesthesia following the principles of Video-Assisted Thoracoscopic Surgery (VATS) with single-lung ventilation in the lateral decubitus position. The extent of lung resection was determined based on the surgeon’s decision, and in cases where VATS was not feasible, the procedure was converted to an open thoracotomy.

The decision regarding medical inoperability and the optimal treatment method was made through a multidisciplinary conference involving a radiologist, thoracic surgeon, pulmonologist, radiation oncologist, and medical oncologist. Medical inoperability was determined based on factors such as advanced age, poor pulmonary function, or significant comorbidities, including cardiovascular or cerebrovascular disease, that rendered surgical resection unsafe or posed a high risk. In particular, there were cases where biopsy and surgical resection were not feasible due to factors such as difficult tumor locations or underlying conditions like interstitial lung disease or cardiac issues. In these cases, PET-CT was essentially performed to evaluate the risk of malignancy, confirm cN0 status, and ensure the absence of distant metastasis, with the interpretation of test results and treatment decisions made through a multidisciplinary conference. Based on these assessments, the multidisciplinary team determined whether SBRT was necessary, even in the absence of tissue confirmation.

All patients received SBRT following a standardized protocol, using immobilization devices and respiratory motion management to ensure precision. Radiation doses varied according to tumor characteristics, with treatments delivered in 1 to 10 fractions, totaling between 28.5 Gy and 80 Gy. The goal was to ensure comprehensive coverage of the planning target volume (PTV) with at least 80% of the prescribed dose. Treatment protocols adhered to recommendations by the American Association of Physicists in Medicine Task Group 101 [9].

### 2.3. CT Image Acquisition and AI-Assisted Image Analysis

All patients underwent chest CT preoperatively using 64-channel multidetector CT scanners. For the assessment of tumor size, type, location, and centrality, preoperative chest CT images were analyzed as references. Four thoracic radiologists (Young Joo Suh, Kyunsun Nam, Na Young Kim, Suji Lee) reviewed the CT image analysis, which was assisted by artificial intelligence (AI)-based computer-aided detection (CAD) software (CT AI-CAD) (AVIEW LCS, v1.1.46.15, Coreline Soft, Seoul, Republic of Korea), to evaluate lung cancer characteristics [10]. The radiologist reviewers had information about the confirmed lung cancer when reviewing the CT AI-CAD results. CT AI-CAD was used as a first reader and the reviewers reviewed the AI-CAD results and corrected the size (the maximal diameter of solid portion on the multi-planar planes), type (solid, part-solid, or non-solid), and lobar location of the CAD-detected malignant nodules if needed. When the CT AI-CAD could not detect the malignant nodule, the reviewers drew the contour of the nodule using semi-automated or manual methods. In addition, the centrality of the malignant nodule and pleural attachment classification were assessed according to the definition proposed by previous studies [11,12]. Specifically, pleural attachment was categorized as follows: no evidence of pleural invasion on CT, pleural contact involving more than one-fourth of the tumor circumference, pleural (or fissural) retraction, pleural tags with thickening of the pleural end, and pleural contact involving less than one-fourth of the tumor circumference or pleural tags without thickening of the pleural end.

### 2.4. Statistical Analysis

A propensity score overlap weighting analysis [13] was performed to balance differences in baseline characteristics between patients who received SBRT and surgery. To calculate the propensity scores (PS) for treatment, we used a multivariable logistic regression model that included the following covariates: age, sex, smoking status (non-smoker or ever-smoker), Eastern Cooperative Oncology Group (ECOG) (0–1 or 2–3), solid tumor diameter, nodule type (solid, part-solid, or non-solid), pleural attachment, centrality (peripheral or central), stage (IA or IB), the square root of age, and the interaction of age and ECOG. We then applied overlap weighting, which assigns weights proportional to the probability of receiving the opposite treatment group (i.e., weight = 1 − PS for patients who received SBRT and weight = PS for those who received surgery, where PS indicates the propensity score for receiving SBRT). This approach down-weights patients with extreme propensity scores (i.e., those with a high probability of receiving SBRT or surgery) and places more weight on those with similar probabilities of receiving either treatment. By targeting patients in clinical equipoise, this method constructs a weighted population that closely mimics a randomized clinical trial [14]. In sensitivity analysis, we used 1:1 nearest neighbor matching with a 0.2 caliper for propensity score [15]. Covariate balance between treatment groups was assessed using absolute standardized mean differences (ASDs), defined as the absolute difference in means or proportions between treatment groups divided by the pooled standard deviation. An ASD < 0.1 was considered indicative of adequately balanced covariates [16]. The primary outcomes were time-to-recurrence and time-to-death from the first date of SBRT or the date of surgery, and patients followed until death or the last follow-up. In the analysis of the first recurrence of lung cancer, death from any cause was treated as a competing risk because death precludes subsequent recurrence. For the first recurrence, we calculated the incidence rate and estimated the subdistribution hazard ratio (sHR) and rate difference (RD), along with their 95% confidence intervals (CIs), overall and within subgroups stratified by risk factors, using Gray’s test for comparison. In the analysis of all-cause mortality, we calculated the incidence rate and estimated the hazard ratio (HR) and RD, with 95% CIs, using the log-rank test and Cox proportional hazards model with robust variance. For solid diameter as a prognostic factor on the first recurrence, we identified the most discriminative cut-off point using the maximally selected rank statistics method, adapted for time-to-event outcomes with competing risks [17]. All tests were 2-sided, and an α level of 0.05 was considered statistically significant. All statistical analyses were performed using R programming language version 4.3.2 (R Foundation for Statistical Computing, Vienna, Austria).

## 3. Results

### 3.1. Study Cohort Characteristics

Baseline patient characteristics are listed in Table 1. Of the total patients analyzed, 216 were included in the SBRT cohort and 1258 in the surgery cohort. The SBRT group had a higher median age (79 years [IQR: 74.0–83.0]) compared to the surgery group (65 years [IQR: 58.0–71.0]), with a greater proportion of males (71.8% vs. 45.5%). The SBRT cohort also had a higher percentage of ever-smokers (61.1% vs. 36.6%) and more patients with a higher ECOG performance status of 2–3 (9.3% vs. 0%). In terms of tumor characteristics, the SBRT group had a larger median solid nodule diameter (18.5 mm [IQR: 12.7–25.4] vs. 13.6 mm [IQR: 6.2–21.3]) and a higher proportion of solid nodules (55.1% vs. 33.2%), while the surgery group had a higher percentage of part-solid nodules (54.5% vs. 42.6%) and non-solid nodules (12.2% vs. 2.3%). In the SBRT group, stage IB was more prevalent than stage IA compared to the surgery group (stage IB: 19.0% vs. 14.9%). After overlap weighting, the baseline characteristics between the SBRT and surgery groups became well balanced, with absolute standardized differences (ASD) all below 0.01 across variables, indicating effective matching in age, sex, smoking status, ECOG, nodule characteristics, and stage (Table 1). Additionally, it was confirmed that the baseline characteristics remained well balanced when propensity score matching was applied (Table S1). Figure S1 illustrates the distribution of propensity scores before and after overlap weighting and matching, respectively. This demonstrates successful adjustment for confounding variables.

In the surgery group, adenocarcinoma was the most common histology, identified in 1147 patients (91.2%), followed by squamous cell carcinoma in 98 patients (7.8%) and other histologies in 13 patients (1%). In contrast, among the SBRT group, adenocarcinoma was confirmed in 71 patients (32.9%), squamous cell carcinoma in 31 patients (14.4%), and other types in 2 patients (0.9%). Approximately half of the SBRT group (112 out of 216 SBRT patients, accounting for 7.6% of the total cohort of 1474 patients) were treated based on multidisciplinary radiologic diagnoses, lacking pathological confirmation despite efforts to obtain tissue samples, primarily due to challenging biopsy location or significant patient comorbidities.

In the SBRT group, the radiotherapy modalities included volumetric modulated arc therapy (VMAT), which was utilized in 90.5% of cases, 3D radiotherapy in 5.5%, and cyberknife in 4%. The median total dose (biologically effective dose with α/β = 10 (BED_10_)) was 112.5 Gy_10_ (IQR, 105.6–150). The median total fraction was 4 (IQR, 4–5) and the median fractional dose was 15 Gy (IQR: 10–15). The median planning target volume (PTV) was 22.9 cm^3^ (IQR, 15.3–37.3).

Among the 1258 patients who underwent surgery, 1226 (97.5%) underwent VATS, while 32 (2.5%) underwent open surgery. Regarding the types of surgical procedures performed, 905 patients (71.9%) underwent lobectomy, 244 patients (19.4%) segmentectomy, 108 patients (8.6%) wedge resection, and one patient (0.1%) pneumonectomy.

### 3.2. Survival Outcomes

**Figure 2:**
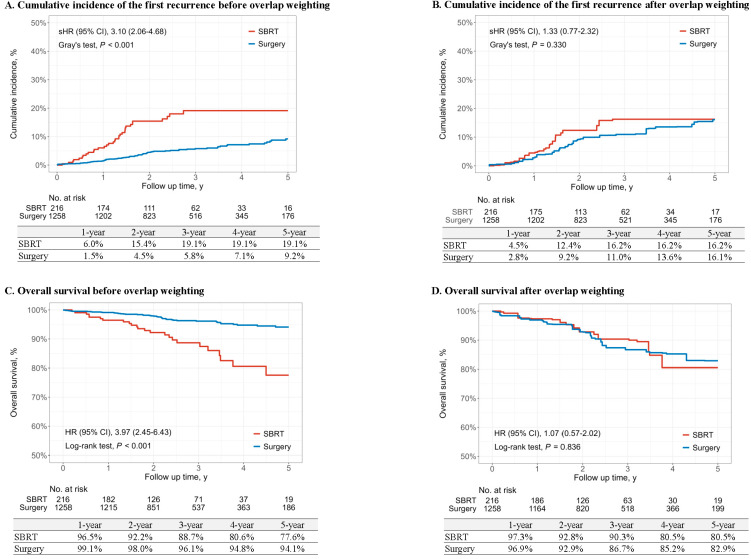
Cumulative incidence of the first recurrence and overall survival of lung cancer. (**A**,**B**) Cumulative incidence of first lung cancer recurrence before (**A**) and after (**B**) overlap weighting. (**C**,**D**) Overall survival before (**C**) and after (**D**) overlap weighting.

During the study period, the first recurrence of lung cancer was observed in 33 of 216 patients (15.3%) in the SBRT group and 74 of 1258 patients (5.9%) in the surgery group (Table 2). All-cause mortality was reported in 26 of 216 patients (12.0%) in the SBRT group and 47 of 1258 patients (3.7%) in the surgery group. Propensity score overlap weighting was applied to estimate the 5-year cumulative incidence of the first recurrence and the 5-year overall survival. After overlap weighting, the 5-year cumulative incidence of recurrence was 16.2% for the SBRT group and 16.1% for the surgery group, with no significant difference (Gray’s test, *p* = 0.330) (Figure 2A,B). The sHR for SBRT was 1.33 (95% CI: 0.77–2.32) compared to surgery, indicating no significant difference in risk (Table 2). Consistent findings were observed when using the propensity score matching method, as shown in Table S2 and Figure S2A.

The 5-year overall survival consistently demonstrated the similarity between the two groups. The 5-year overall survival was not statistically different between SBRT (80.5%) and surgery (82.9%) after overlap weighting (*p* = 0.836). The hazard ratio was 1.07 (95% CI: 0.57–2.02), with the surgery group as the reference, indicating no significant difference (Table 2 and Figure 2C,D). Similarly, propensity score matching confirmed no significant difference in the 5-year overall survival, aligning with the overlap weighting result (Table S2, Figure S2B).

### 3.3. Subgroup Analyses Stratified by Risk Factors

**Figure 3:**
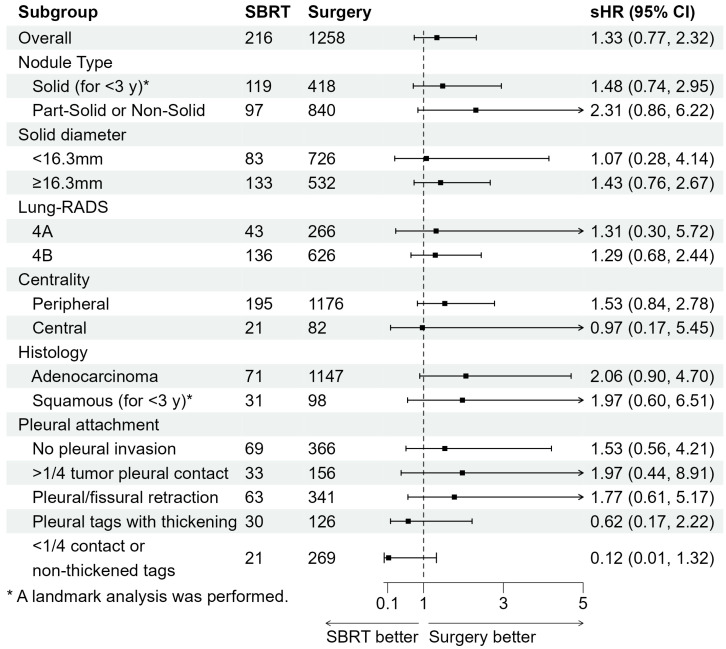
Subgroup analyses stratified by risk factors for the first recurrence of lung cancer after overlap weighting. The overlap weights were re-created for each subgroup analysis. If the proportional hazards assumption is violated, a landmark analysis is performed. Abbreviations: SBRT, stereotactic body radiotherapy; sHR, subdistribution hazard ratio; CI, confidence interval.

Subgroup analyses, stratified by factors such as nodule type, solid nodule diameter, centrality, histology, and pleural attachment are summarized Figure 3 and Table S3. These analyses consistently demonstrated similar recurrence risks between the SBRT and surgery groups across all subgroups. Specifically, sHR remained comparable across nodule types (solid vs. part-solid/non-solid), diameters, centrality, pleural attachment, and tumor histology, with no significant variation (Figure S3). These findings suggest that, after overlap weighting, the recurrence risks between the SBRT and surgery show no meaningful differences in stage I NSCLC, regardless of subgroup characteristics.

**Figure 4:**
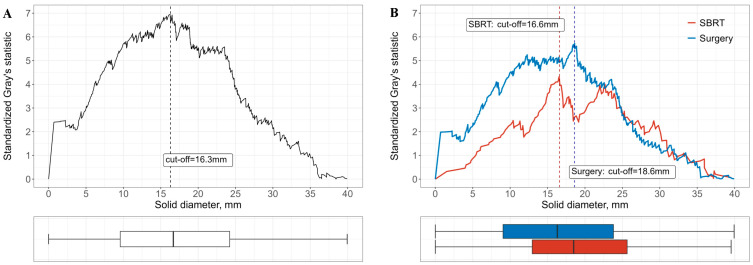
Plot of standardized Gray’s statistics against solid diameters, and box plot of solid diameters. (**A**) Analysis based on the overall study population. (**B**) Subgroup analysis stratified by treatment modality. The vertical line represents the value estimated as the optimal cutoff for the solid diameter.

In addition, standardized Gray’s statistics identified solid tumor diameter associated with a significant increase in recurrence (Figure 4). The cutoff value for recurrence risks in the total cohort was 16.3 mm, while subgroup-specific cutoff value was 16.6 mm for SBRT group and 18.6 mm for the surgery group.

### 3.4. Mortality and Cause of Death Within 90 Days After Treatment

There were two deaths in the SBRT group and five deaths in the surgery group within 90 days of post-treatment. In the SBRT group, an 83-year-old male patient died 63 days after completing radiation therapy, and a 77-year-old male patient died 80 days after treatment. Both deaths were attributed to the progression of preexisting comorbidities, with no evidence of a causal relationship with the SBRT. In the surgery group, there were five deaths. Two patients died due to postoperative complications. One patient, who was discharged without notable complications following lung cancer surgery, passed away at a hospital outside our facility from unknown causes. The other two deaths were caused by cardiac arrest and aortic dissection. Detailed causes of death within 90 days post-treatment are summarized in Table S4.

## 4. Discussion

In this study, we found no significant differences in the risk of recurrence or overall survival between patients with stage I lung cancer treated with SBRT and those who underwent surgical resection, after applying propensity score overlap weighting. Subgroup analysis across various tumor characteristics consistently demonstrated comparable treatment outcomes between SBRT and surgery.

Previous studies have investigated the comparative outcomes of surgery and SBRT for stage I NSCLC, yielding mixed and often controversial results. Some research has suggested that overall survival following surgery and SBRT is comparable, though these findings often come from studies with limitations such as small sample sizes and short follow-up periods [18,19,20]. On the other hand, other studies have demonstrated the superiority of surgery over SBRT [21,22,23]. Given these varying outcomes, current guidelines endorse SBRT as an appropriate alternative for patients with stage I NSCLC who are not surgical candidates, emphasizing the importance of personalized treatment strategies tailored to individual patient conditions [5,24].

Because blinded randomized controlled trials (RCTs) remain challenging to conduct in this context, many studies have relied on methods like propensity score matching (PSM) to adjust for confounding factors [22,23]. A key difference from previous studies is that they primarily matched patients based on demographic and basic clinical factors such as age, sex, performance status, histology, and tumor size [25,26,27]. In contrast, our study compares the outcomes of surgery and SBRT in stage I lung cancer patients using propensity score overlap weighting, incorporating advanced CT-based AI CAD technology for detailed tumor analysis, including nodule type, size, centrality, and pleural attachment. We used propensity score overlap weighting to address substantial differences in patient characteristics between the treatment groups.

In our study, patients with better clinical condition were more likely to be assigned to the surgery group, whereas those with poorer clinical status tended to receive SBRT. This clinical decision-making pattern led to confounding by indication, resulting in substantial baseline imbalances between the treatment groups. Given this strong selection bias, PSM may not adequately balance covariates or may result in considerable loss of sample size due to limited overlap in propensity scores. To address this selection bias, we applied overlap weighting to our primary analysis for comparing treatment effects. This approach emphasizes patients who have a similar probability of receiving either treatment (i.e., those in clinical equipoise). As a result, even when the original cohort exhibits substantial differences between treatment groups, covariate balance can be improved in the weighted cohort. We also conducted a sensitivity analysis using PSM to evaluate the robustness of our primary analysis (Table S1).

After adjusting for differences in measured covariates between SBRT and surgery treatments using overlap weighting, no significant association was observed between the treatment and outcomes in the target population at clinical equipoise for the treatment decision. These findings remained consistent even in subgroup analyses stratified according to radiologic tumor characteristics identified with the assistance of CT AI-CAD, where the overall outcomes for stage I lung cancer were not statistically significant. In this study, the use of both propensity score weighting and propensity score matching methods consistently demonstrated similar results, further supporting the robustness of the findings.

We noted that in our study, a solid tumor diameter greater than 16.6 mm for SBRT and 18.6 mm for surgery was significantly associated with higher recurrence rates after curative treatment. Early-stage lung cancer is often detected as smaller lung nodules, and patients undergo periodic chest CT scans until significant changes, such as an increase in size before prompt biopsy or treatment. This recurrence risk cut-off value in solid diameter could play a crucial role in determining the optimal timing for proactive diagnostic and therapeutic interventions during imaging follow-up.

In this study, about half of the patients in the SBRT group (112 out of 216, accounting for 7.6% of the total cohort of 1474 patients) received treatment without histologic confirmation, despite efforts to acquire tissue samples. This was primarily due to small lesion size, difficult tumor locations, or underlying lung conditions—such as emphysema or interstitial lung disease—that made biopsy unsafe due to high complication risk. Moreover, many nodules were subsolid or ground-glass opacities (GGOs), which are known to have low diagnostic yield with biopsy, further limiting feasibility. Although the absence of pathological confirmation may be viewed as a limitation, it reflects real-world clinical scenarios in which treatment decisions are often based on multidisciplinary radiologic assessment. In this setting, the integration of AI into the diagnostic process has played a key role in enhancing the accuracy and consistency of lung nodule classification, enabling clinicians to determine the necessity of histologic confirmation and treatment by predicting the malignancy of the nodule. Moreover, AI-based predictive models have the potential to assist multidisciplinary teams in estimating prognosis and guiding individualized therapeutic decisions, as demonstrated in recent study [28].

To further support the safety of SBRT, particularly in patients for whom surgery or biopsy is not feasible, we previously analyzed 271 patients with early-stage NSCLC (276 lesions) who underwent SBRT at our institution between 2012 and 2022, focusing on recurrence patterns and radiation pneumonitis, the most representative complication associated with SBRT [29]. In this study, larger tumor size (*p* < 0.001) and a higher solid-to-total tumor ratio (*p* = 0.028) were significantly associated with increased risk of local recurrence. Symptomatic radiation pneumonitis occurred in 7.2% of lesions and was also significantly associated with larger solid tumor size (*p* = 0.050). These findings suggest that SBRT can be performed safely with lower risks of recurrence and radiation pneumonitis in patients with early-stage NSCLC.

This study has several limitations. First, although the data spans a decade and involves a multidisciplinary approach, it is derived from a single-center, retrospective analysis, which limits the generalizability of our findings. Second, unlike randomized clinical trials, cohort studies are inherently susceptible to selection bias and confounding from both known and unknown variables [30]. To address this, we applied propensity score overlap weighting to adjust for available prognostic variables. However, unmeasured confounders, such as the lack of histological confirmation in some patients, could introduce bias, potentially affecting the validity of the findings. Lastly, while we assessed overall mortality and 90-day treatment-related deaths, quality of life outcomes were not evaluated, which remains an important aspect for future research. Despite these limitations, our findings offer valuable insights that can inform the design of future prospective trials and provide additional support for clinical decision-making in the absence of definitive evidence from randomized trials.

## 5. Conclusions

In conclusion, this study demonstrates that after propensity score overlap weighting and adjustment for patient and tumor characteristics, SBRT and surgery yield comparable treatment outcomes in stage I NSCLC. In the current context where early-stage lung cancer is increasingly detected in elderly patients or those with various underlying comorbidities, this study may help optimize the timing and approach to safe and effective treatments for stage I lung cancer patients.

## Figures and Tables

**Table 1:** Baseline characteristics before and after overlap weighting.

Characteristic	Before Overlap Weighting			After Overlap Weighting		
	SBRT (*n* = 216)	Surgery (*n* = 1258)	ASD	SBRT (*n* = 216)	Surgery (*n* = 1258)	ASD
Age	79.0 (74.0, 83.0)	65.0 (58.0, 71.0)	1.630	75.0 (69.0, 79.0)	75.0 (71.0, 78.0)	<0.001
Sex			0.552			<0.001
Male	155 (71.8%)	573 (45.5%)		145 (67.2%)	846 (67.2%)	
Female	61 (28.2%)	685 (54.5%)		71 (32.8%)	412 (32.8%)	
Smoking			0.505			<0.001
Non-smoker	84 (38.9%)	797 (63.4%)		92 (42.6%)	536 (42.6%)	
Ever-smoker	132 (61.1%)	461 (36.6%)		124 (57.4%)	722 (57.4%)	
ECOG			0.452			<0.001
0~1	196 (90.7%)	1258 (100.0%)		216 (100.0%)	1258 (100.0%)	
2~3	20 (9.3%)	0 (0.0%)		0 (0.0%)	0 (0.0%)	
Solid diameter	18.5 (12.7, 25.4)	13.6 (6.2, 21.3)	0.483	17.5 (11.5, 23.8)	17.8 (11.2, 24.7)	<0.001
Nodule type			0.541			<0.001
Solid	119 (55.1%)	418 (33.2%)		111 (51.6%)	649 (51.6%)	
Part-Solid	92 (42.6%)	686 (54.5%)		99 (45.8%)	576 (45.8%)	
Non-Solid	5 (2.3%)	154 (12.2%)		6 (2.6%)	33 (2.6%)	
Pleural attachment			0.337			<0.001
No pleural invasion	69 (31.9%)	366 (29.1%)		69 (32.1%)	404 (32.1%)	
>1/4 tumor pleural contact	33 (15.3%)	156 (12.4%)		30 (14.0%)	176 (14.0%)	
Pleural/fissural retraction	63 (29.2%)	341 (27.1%)		63 (29.0%)	364 (29.0%)	
Pleural tags with thickening	30 (13.9%)	126 (10.0%)		28 (12.9%)	163 (12.9%)	
<1/4 contact or non-thickened tags	21 (9.7%)	269 (21.4%)		26 (12.1%)	152 (12.1%)	
Centrality			0.117			<0.001
Peripheral	195 (90.3%)	1176 (93.5%)		197 (91.3%)	1149 (91.3%)	
Central	21 (9.7%)	82 (6.5%)		19 (8.7%)	109 (8.7%)	
Stage			0.108			<0.001
IA	175 (81.0%)	1070 (85.1%)		176 (81.4%)	1024 (81.4%)	
IB	41 (19.0%)	188 (14.9%)		40 (18.6%)	234 (18.6%)	

Abbreviations: SBRT, stereotactic body radiotherapy; ASD, absolute standardized difference. Data are reported as medians (IQRs) for continuous variables and numbers (percentages) for categorical variables.

**Table 2:** Risk of the first recurrence of lung cancer and the 5-year overall survival by SBRT or surgery among patients with stage I lung cancer before and after overlap weighting.

Variable	Before Overlap Weighting			After Overlap Weighting		
	SBRT	Surgery	*p*-Value	SBRT	Surgery	*p*-Value
**First recurrence of lung cancer**						
No. of events/No. of patients	33/216	74/1258	-	27/216	138/1258	-
Time to event, median (IQR), y	2.03 (1.34, 3.21)	2.50 (1.54, 4.10)	-	2.04 (1.45, 3.10)	2.42 (1.49, 4.04)	-
IR, per 100 person-years	6.42	1.96	-	5.36	3.83	-
RD (95% CI)	4.46 (2.22, 6.70)	0 [Reference]	<0.001	1.54 (−0.58, 3.65)	0 [Reference]	0.156
sHR (95% CI)	3.10 (2.06, 4.68)	1 [Reference]	<0.001	1.33 (0.77, 2.32)	1 [Reference]	0.310
**Overall survival**						
No. of events/No. of patients	26/216	47/1258	-	23/216	144/1258	-
Time to event, median (IQR), y	2.35 (1.49, 3.33)	2.54 (1.56, 4.26)	-	2.30 (1.65, 3.21)	2.49 (1.52, 4.29)	-
IR, per 100 person-years	4.70	1.21	-	4.18	3.78	-
RD (95% CI)	3.48 (1.65, 5.32)	0 [Reference]	<0.001	0.40 (−1.43, 2.23)	0 [Reference]	0.668
HR (95% CI)	3.97 (2.45, 6.43)	1 [Reference]	<0.001	1.07 (0.57, 2.02)	1 [Reference]	0.835

Abbreviations: SBRT, stereotactic body radiotherapy; IR, incidence rate; RD, rate difference; HR, hazard ratio; sHR, subdistribution hazard ratio; IQR, interquartile range; CI, confidence interval. Median time-to-event period was calculated using the event time for patients experienced events and the last follow-up time for those who did not.

## Data Availability

The datasets generated and/or analyzed during this study are available from the corresponding author upon reasonable request.

## Supplementary Materials

The following supporting information can be downloaded at: https://www.mdpi.com/article/10.3390/cancers17122015/s1, Figure S1. Distribution of propensity scores before and after overlap weighting or matching; Figure S2. Cumulative incidence of the first recurrence and overall survival of lung cancer after matching; Figure S3. Subgroup analyses stratified by risk factors for the first recurrence of lung cancer after overlap weighting; Table S1. Baseline characteristics before and after propensity score matching; Table S2. Risk of the first recurrence of lung cancer and the 5-year overall survival by SBRT or surgery among patients with stage 1 lung cancer before and after propensity score matching; Table S3. Subgroup analyses stratified by risk factors for the first recurrence of lung cancer; Table S4. Mortality and cause of death within 90 days after treatment in SBRT and Surgery Groups.

## Abbreviations

NSCLC, non-small cell lung cancer; SBRT, stereotactic body radiotherapy; ECOG, Eastern Cooperative Oncology Group; PET-CT, Positron Emission Tomography-Computed Tomography; VATS, Video-Assisted Thoracoscopic Surgery; AI, artificial intelligence; CAD, computer-aided detection; sHR, subdistribution hazard ratio; CI, confidence interval; RD, rate difference; ASD, Absolute standardized mean differences.
