# Supplementary material for: Comparison of Stereotactic Body Radiotherapy and Surgery for Stage I Lung Cancer: A Multidisciplinary Cohort Study Utilizing Propensity Score Overlap Weighting and AI-Based CT Imaging Analysis
Source: Cancers (Basel). 2025 Jun 17;17(12):2015. doi: 10.3390/cancers17122015 (PMC12190411; doi:10.3390/cancers17122015)
Supplement: Supplementary file 1 [file cancers-17-02015-s001.zip › SupplementaryFigures.pdf]

Supplementary Figures

Figure S1. Distribution of propensity scores before and after overlap weighting or matching

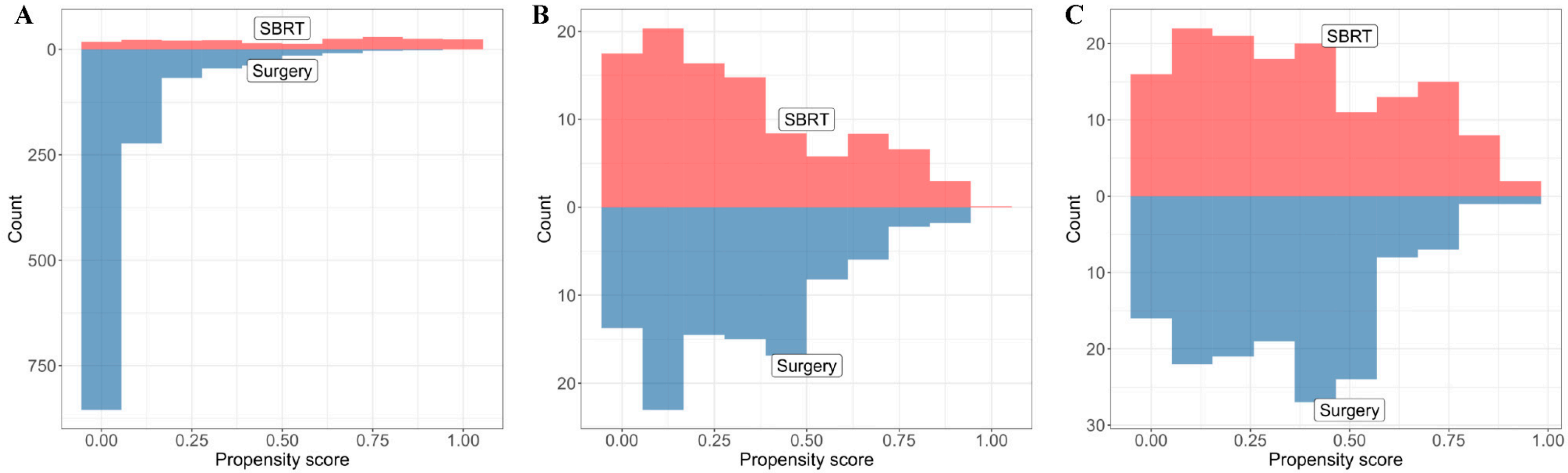

(A) Before overlap weighting, (B) After overlap weighting, (C) After matching.

Figure S2. Cumulative incidence of the first recurrence and overall survival of lung cancer after matching.

A. Cumulative incidence of the first recurrence after matching

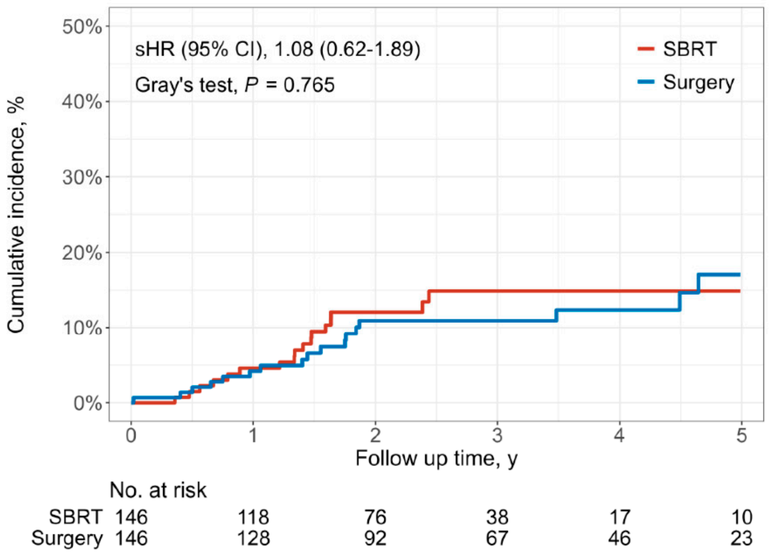

	1-year	2-year	3-year	4-year	5-year
SBRT	4.6%	12.1%	14.9%	14.9%	14.9%
Surgery	4.2%	10.9%	10.9%	12.3%	17.1%

B. Overall survival after matching

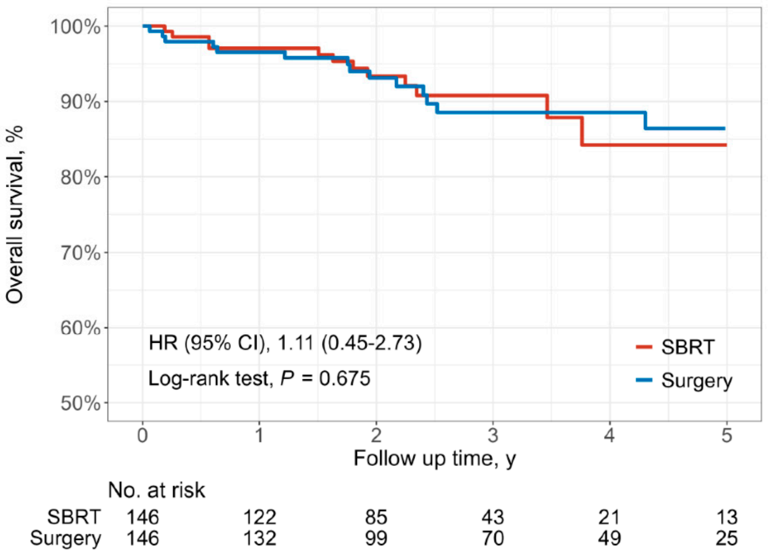

	1-year	2-year	3-year	4-year	5-year
SBRT	97.0%	93.4%	90.8%	84.2%	84.2%
Surgery	96.6%	93.1%	88.5%	88.5%	86.4%

(A) First recurrence of lung cancer, (B) Overall survival.

Figure S3. Subgroup analyses stratified by risk factors for the first recurrence of lung cancer after overlap weighting.

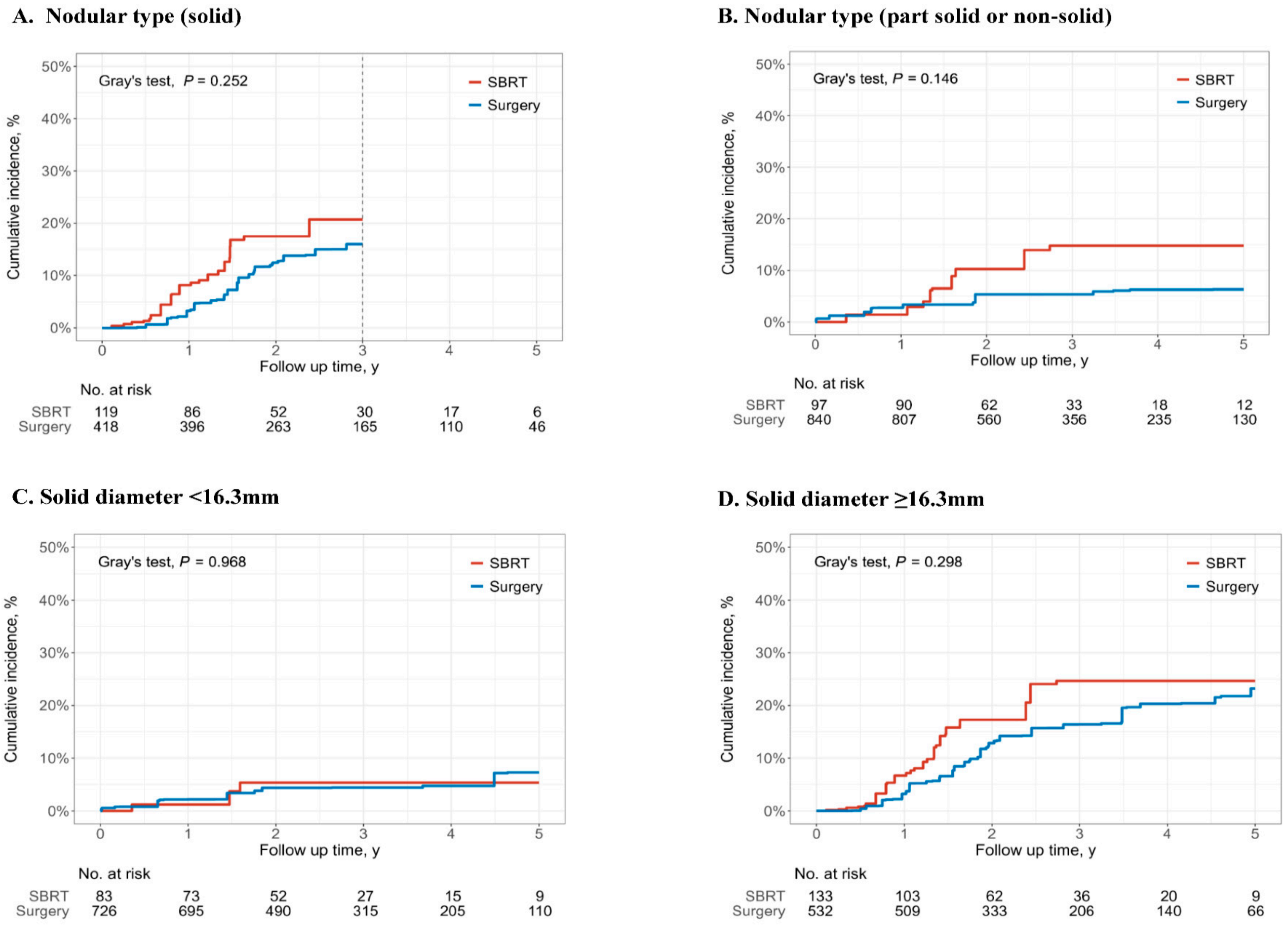

E. LungRADS (4A)

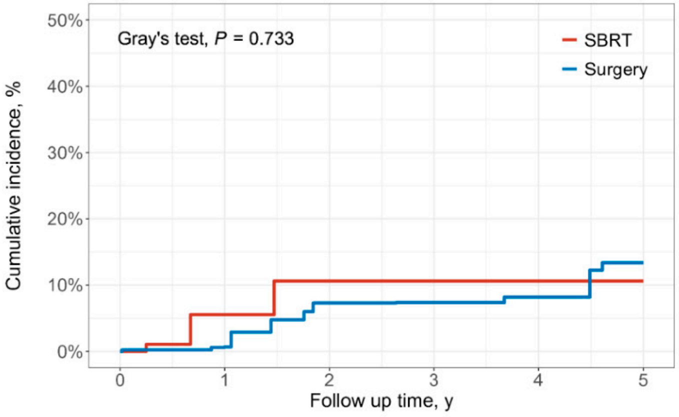

No. at risk

	0	1	2	3	4	5
SBRT	43	33	24	15	9	5
Surgery	266	256	166	113	83	41

F. LungRADS (4B)

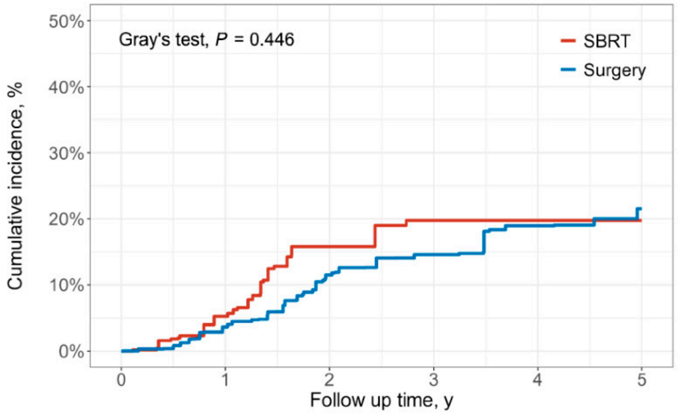

No. at risk

	0	1	2	3	4	5
SBRT	136	110	66	37	19	8
Surgery	626	593	404	258	168	86

G. Peripheral

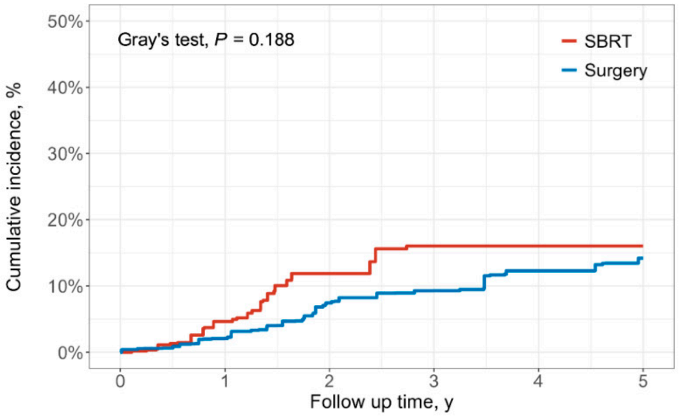

No. at risk

	0	1	2	3	4	5
SBRT	195	158	102	56	29	14
Surgery	1176	1123	782	487	317	167

H. Central

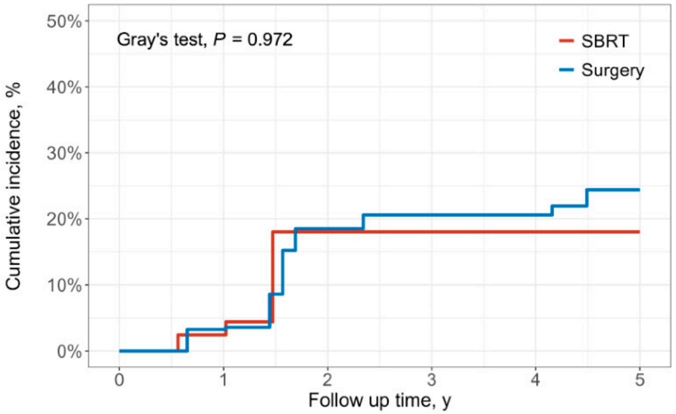

No. at risk

	0	1	2	3	4	5
SBRT	21	18	12	7	6	4
Surgery	82	80	42	35	28	10

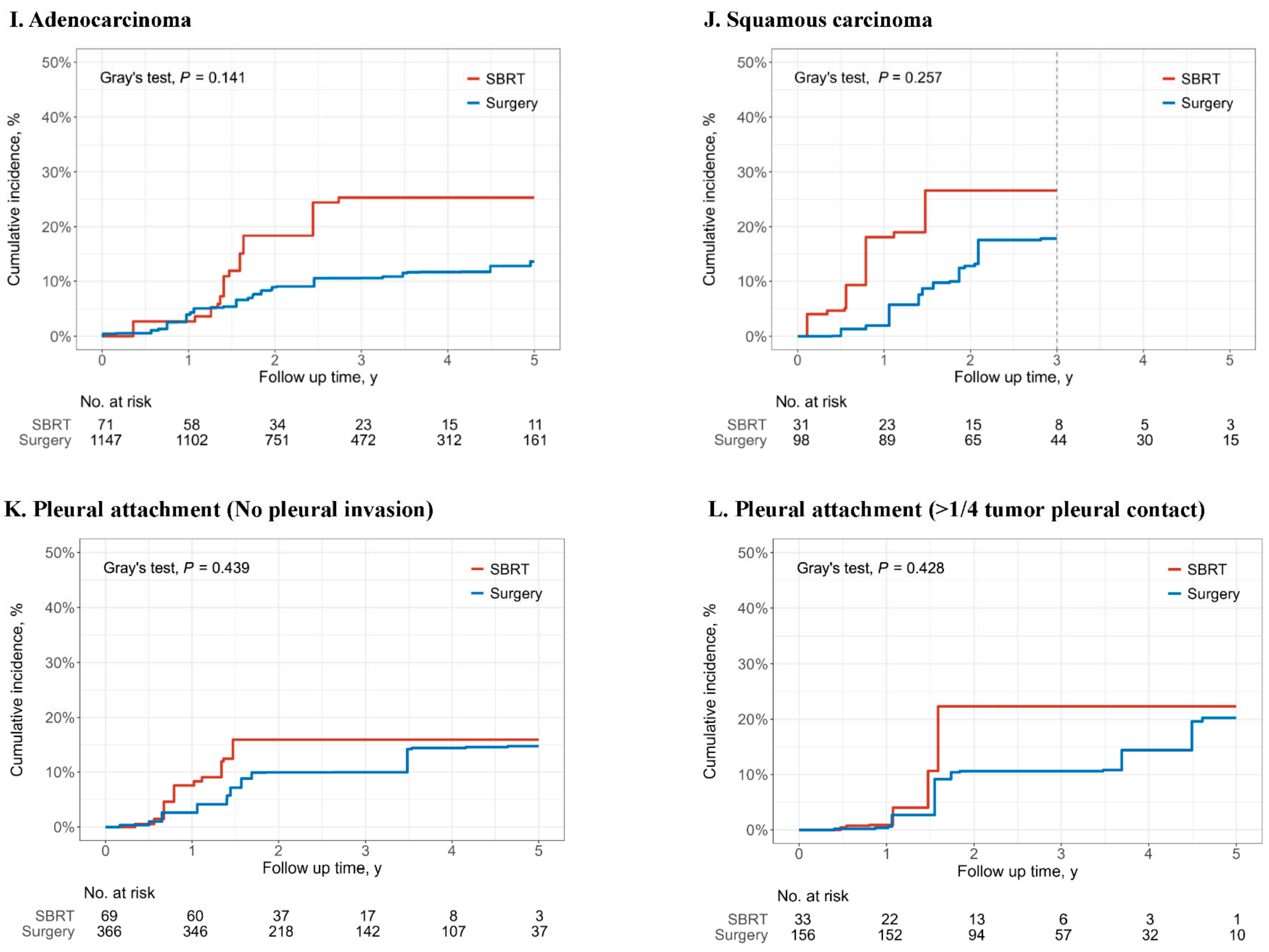
I. Adenocarcinoma
Gray's test, P = 0.141
SBRT
Surgery
Cumulative incidence, %
Follow up time, y
No. at risk
SBRT 71 58 34 23 15 11
Surgery 1147 1102 751 472 312 161
J. Squamous carcinoma
Gray's test, P = 0.257
SBRT
Surgery
Cumulative incidence, %
Follow up time, y
No. at risk
SBRT 31 23 15 8 5 3
Surgery 98 89 65 44 30 15
K. Pleural attachment (No pleural invasion)
Gray's test, P = 0.439
SBRT
Surgery
Cumulative incidence, %
Follow up time, y
No. at risk
SBRT 69 60 37 17 8 3
Surgery 366 346 218 142 107 37
L. Pleural attachment (>1/4 tumor pleural contact)
Gray's test, P = 0.428
SBRT
Surgery
Cumulative incidence, %
Follow up time, y
No. at risk
SBRT 33 22 13 6 3 1
Surgery 156 152 94 57 32 10

M. Pleural attachment (Pleural/fissural retraction)

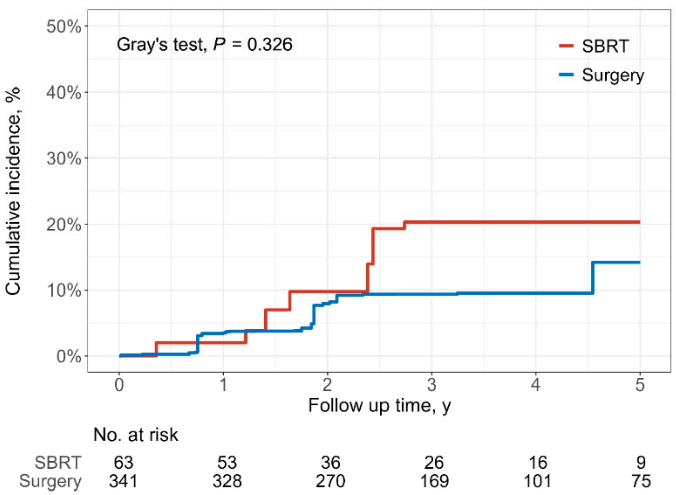

N. Pleural attachment (Pleural tags with thickening)

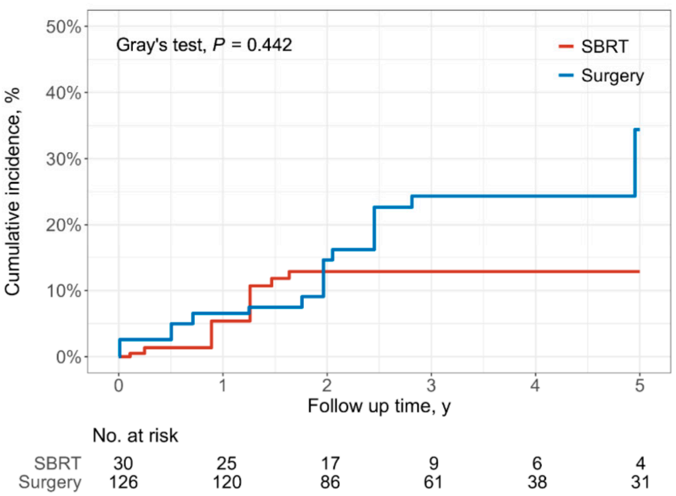

(A) Nodule type = Solid, (B) Nodule type = Part or Non-Solid, (C) Solid diameter <16.3mm, (D) Solid diameter ≥16.3mm, (E) LungRADS = 4A, (F) LungRADS = 4B, (G) Peripheral, (H) Central, (I) Histology = Adenocarcinoma, (J) Histology = Squamous, (K) Pleural attachment = No pleural invasion, (L) Pleural attachment = >1/4 tumor pleural contact, (M) Pleural attachment = Pleural/fissural retraction, (N) Pleural attachment = Pleural tags with thickening.
