# Supplementary material for: Comparison of Stereotactic Body Radiotherapy and Surgery for Stage I Lung Cancer: A Multidisciplinary Cohort Study Utilizing Propensity Score Overlap Weighting and AI-Based CT Imaging Analysis
Source: Cancers (Basel). 2025 Jun 17;17(12):2015. doi: 10.3390/cancers17122015 (PMC12190411; doi:10.3390/cancers17122015)
Supplement: Supplementary file 1 [file cancers-17-02015-s001.zip › SupplementaryTables.pdf]

Supplementary Tables

Table S1. Baseline characteristics before and after propensity score matching

Characteristic	Before matching			After matching		
	SBRT (n=216)	Surgery (n=1,258)	ASD	SBRT (n=146)	Surgery (n=146)	ASD
Age	79.0 (74.0, 83.0)	65.0 (58.0, 71.0)	1.630	77.0 (71.0, 80.0)	76.0 (73.0, 78.0)	0.096
Sex			0.552			0.030
Male	155 (71.8%)	573 (45.5%)		100 (68.5%)	102 (69.9%)	
Female	61 (28.2%)	685 (54.5%)		46 (31.5%)	44 (30.1%)	
Smoking			0.505			0.042
Non-smoker	84 (38.9%)	797 (63.4%)		60 (41.1%)	63 (43.2%)	
Ever-smoker	132 (61.1%)	461 (36.6%)		86 (58.9%)	83 (56.8%)	
ECOG			0.452			<0.001
0~1	196 (90.7%)	1,258 (100.0%)		146 (100.0%)	146 (100.0%)	
2~3	20 (9.3%)	0 (0.0%)		0 (0.0%)	0 (0.0%)	
Solid diameter	18.5 (12.7, 25.4)	13.6 (6.2, 21.3)	0.483	17.9 (12.6, 23.9)	17.1 (11.2, 25.3)	0.001
Nodule type			0.541			0.028
Solid	119 (55.1%)	418 (33.2%)		75 (51.4%)	77 (52.7%)	
Part-Solid	92 (42.6%)	686 (54.5%)		68 (46.6%)	66 (45.2%)	
Non-Solid	5 (2.3%)	154 (12.2%)		3 (2.1%)	3 (2.1%)	
Pleural attachment			0.337			0.046
No pleural invasion	69 (31.9%)	366 (29.1%)		46 (31.5%)	45 (30.8%)	
>1/4 tumor pleural contact	33 (15.3%)	156 (12.4%)		23 (15.8%)	23 (15.8%)	
Pleural/fissural retraction	63 (29.2%)	341 (27.1%)		43 (29.5%)	43 (29.5%)	
Pleural tags with thickening	30 (13.9%)	126 (10.0%)		17 (11.6%)	19 (13.0%)	
<1/4 contact or non-thickened tags	21 (9.7%)	269 (21.4%)		17 (11.6%)	16 (11.0%)	

Centrality			0.117			0.024
Peripheral	195 (90.3%)	1,176 (93.5%)		133 (91.1%)	134 (91.8%)	
Central	21 (9.7%)	82 (6.5%)		13 (8.9%)	12 (8.2%)	
Stage			0.108			0.053
IA	175 (81.0%)	1,070 (85.1%)		117 (80.1%)	120 (82.2%)	
IB	41 (19.0%)	188 (14.9%)		29 (19.9%)	26 (17.8%)	

Abbreviations: SBRT, stereotactic body radiotherapy; ASD, absolute standardized difference.

Data are reported as medians (IQRs) for continuous variables and numbers (percentages) for categorical variables.

Table S2. Risk of the first recurrence of lung cancer and the 5-year overall survival by SBRT or surgery among patients with stage 1 lung cancer before and after propensity score matching.

Variable	Before matching			After matching		
	SBRT	Surgery	p-value	SBRT	Surgery	p-value
**First recurrence of lung cancer**						
No. of events/No. of patients	33/216	74/1258	-	17/146	18/146	-
Time to event, median (IQR), y	2.03 (1.34, 3.21)	2.50 (1.54, 4.10)	-	2.03 (1.44, 3.14)	2.59 (1.45, 4.41)	-
IR, per 100 person-years	6.42	1.96	-	4.98	4.08	-
RD (95% CI)	4.46 (2.22, 6.70)	0 [Reference]	<0.001	0.90 (-2.13, 3.93)	0 [Reference]	0.560
sHR (95% CI)	3.10 (2.06, 4.68)	1 [Reference]	<0.001	1.08 (0.62, 1.89)	1 [Reference]	0.773
**Overall survival**						
No. of events/No. of patients	26/216	47/1258	-	14/146	15/146	-
Time to event, median (IQR), y	2.35 (1.49, 3.33)	2.54 (1.56, 4.26)	-	2.28 (1.63, 3.22)	2.81 (1.66, 4.48)	-
IR, per 100 person-years	4.70	1.21	-	3.82	3.23	-
RD (95% CI)	3.48 (1.65, 5.32)	0 [Reference]	<0.001	0.59 (-1.99, 3.17)	0 [Reference]	0.654
HR (95% CI)	3.97 (2.45, 6.43)	1 [Reference]	<0.001	1.11 (0.45, 2.73)	1 [Reference]	0.819

Abbreviations: SBRT, stereotactic body radiotherapy; IR, incidence rate; RD, rate difference; HR, hazard ratio; sHR, subdistribution hazard ratio; IQR, interquartile range; CI, confidence interval. Sensitivity analysis was performed using 1:1 propensity score matching.
Median time-to-event period was calculated using the event time for patients experienced events and the last follow-up time for those who did not.

Table S3. Subgroup analyses stratified by risk factors for the first recurrence of lung cancer

Variable	Before overlap weighting			After overlap weighting		
	SBRT	Surgery	p-value	SBRT	Surgery	p-value
**Nodule type**						
**Solid**						
No. of events/No. of patients	24/119	50/418	-	20/119	69/418	-
Time to event, median (IQR), y	1.80 (0.80, 2.99)	2.45 (1.53, 4.05)	-	1.93 (1.41, 2.68)	2.46 (1.47, 4.04)	-
IR, per 100 person-years	9.61	4.11	-	7.63	5.76	-
RD (95% CI)	5.51 (1.50, 9.52)	0 [Reference]	0.007	1.87 (-1.76, 5.50)	0 [Reference]	0.314
sHR (95% CI), For <3 y*	2.62 (1.58, 4.35)	1 [Reference]	<0.001	1.48 (0.74, 2.95)	1 [Reference]	0.268
**Part-Solid or Non-Solid**						
No. of events/No. of patients	9/97	24/840	-	11/97	44/840	-
Time to event, median (IQR), y	2.40 (1.73, 3.33)	2.52 (1.55, 4.10)	-	2.20 (1.49, 3.18)	2.41 (1.52, 4.07)	-
IR, per 100 person-years	3.41	0.94	-	4.43	1.80	-
RD (95% CI)	2.47 (0.21, 4.72)	0 [Reference]	0.032	2.63 (-0.08, 5.33)	0 [Reference]	0.057
sHR (95% CI)	3.50 (1.64, 7.47)	1 [Reference]	0.001	2.31 (0.86, 6.22)	1 [Reference]	0.098
**Solid diameter†**						
**<16.3mm**						
No. of events/No. of patients	3/83	22/726	-	4/83	33/726	-
Time to event, median (IQR), y	2.40 (1.64, 3.27)	2.57 (1.55, 4.11)	-	2.33 (1.61, 3.17)	2.48 (1.50, 4.04)	-
IR, per 100 person-years	1.37	1	-	1.84	1.57	-
RD (95% CI)	0.37 (-1.24, 1.97)	0 [Reference]	0.652	0.27 (-1.64, 2.18)	0 [Reference]	0.784
sHR (95% CI)	1.29 (0.39, 4.28)	1 [Reference]	0.678	1.07 (0.28, 4.14)	1 [Reference]	0.921
**≥16.3mm**						
No. of events/No. of patients	30/133	52/532	-	24/133	86/532	-
Time to event, median (IQR), y	1.93 (1.07, 3.15)	2.42 (1.54, 4.09)	-	1.96 (1.36, 3.08)	2.24 (1.43, 4.15)	-

IR, per 100 person-years	10.18	3.3	-	8.25	5.58	-
RD (95% CI)	6.87 (3.12, 10.62)	0 [Reference]	<0.001	2.67 (-0.84, 6.17)	0 [Reference]	0.136
sHR (95% CI)	2.96 (1.88, 4.65)	1 [Reference]	<0.001	1.43 (0.76, 2.67)	1 [Reference]	0.268
**findings.finalLungRADS**						
**4A**						
No. of events/No. of patients	3/43	13/266	-	4/43	23/266	-
Time to event, median (IQR), y	2.03 (1.06, 3.32)	2.49 (1.52, 4.51)	-	2.02 (1.47, 2.91)	2.98 (1.57, 4.92)	-
IR, per 100 person-years	2.91	1.60	-	3.96	2.58	-
RD (95% CI)	1.32 (-2.09, 4.73)	0 [Reference]	0.449	1.38 (-2.70, 5.46)	0 [Reference]	0.507
sHR (95% CI)	1.73 (0.49, 6.14)	1 [Reference]	0.397	1.31 (0.30, 5.72)	1 [Reference]	0.716
**4B**						
No. of events/No. of patients	27/136	55/626	-	21/136	92/626	-
Time to event, median (IQR), y	1.96 (1.22, 3.17)	2.49 (1.54, 4.10)	-	2.06 (1.41, 3.10)	2.19 (1.40, 3.83)	-
IR, per 100 person-years	8.88	2.94	-	6.89	5.32	-
RD (95% CI)	5.94 (2.50, 9.38)	0 [Reference]	0.001	1.58 (-1.57, 4.72)	0 [Reference]	0.326
sHR (95% CI)	2.85 (1.79, 4.52)	1 [Reference]	<0.001	1.29 (0.68, 2.44)	1 [Reference]	0.429
**Centrality**						
**Peripheral**						
No. of events/No. of patients	30/195	65/1176	-	24/195	110/1176	-
Time to event, median (IQR), y	2.03 (1.34, 3.21)	2.51 (1.56, 4.08)	-	2.06 (1.44, 3.16)	2.39 (1.50, 4.01)	-
IR, per 100 person-years	6.52	1.84	-	5.23	3.33	-
RD (95% CI)	4.68 (2.31, 7.06)	0 [Reference]	<0.001	1.90 (-0.28, 4.08)	0 [Reference]	0.088
sHR (95% CI)	3.35 (2.17, 5.16)	1 [Reference]	<0.001	1.53 (0.84, 2.78)	1 [Reference]	0.160
**Central**						
No. of events/No. of patients	3/21	9/82	-	3/21	16/82	-
Time to event, median (IQR), y	2.20 (1.47, 3.31)	1.97 (1.47, 4.26)	-	1.92 (1.65, 2.20)	3.30 (1.51, 5.68)	-

IR, per 100 person-years	5.58	3.79	-	7.69	5.33	-
RD (95% CI)	1.79 (-5.00, 8.57)	0 [Reference]	0.605	2.36 (-6.30, 11.02)	0 [Reference]	0.593
sHR (95% CI)	1.39 (0.36, 5.37)	1 [Reference]	0.633	0.97 (0.17, 5.45)	1 [Reference]	0.972
**Histology**						
**Adenocarcinoma**						
No. of events/No. of patients	13/71	56/1147	-	12/71	117/1147	-
Time to event, median (IQR), y	1.96 (1.26, 3.49)	2.50 (1.54, 4.08)	-	1.84 (1.24, 3.16)	2.33 (1.37, 3.98)	-
IR, per 100 person-years	7.34	1.63	-	7.51	3.65	-
RD (95% CI)	5.71 (1.70, 9.72)	0 [Reference]	0.005	3.85 (-0.49, 8.20)	0 [Reference]	0.082
sHR (95% CI)	4.38 (2.37, 8.11)	1 [Reference]	<0.001	2.06 (0.90, 4.70)	1 [Reference]	0.085
**Squamous**						
No. of events/No. of patients	9/31	17/98	-	7/31	25/98	-
Time to event, median (IQR), y	1.77 (0.68, 2.82)	2.57 (1.48, 4.51)	-	1.93 (0.79, 2.63)	2.46 (1.74, 4.36)	-
IR, per 100 person-years	13.82	5.76	-	10.69	8.38	-
RD (95% CI)	8.06 (-1.38, 17.49)	0 [Reference]	0.094	2.31 (-6.23, 10.85)	0 [Reference]	0.596
sHR (95% CI), For <3 y*	2.76 (1.19, 6.44)	1 [Reference]	0.019	1.97 (0.60, 6.51)	1 [Reference]	0.264
**Pleural attachment**						
**No pleural invasion**						
No. of events/No. of patients	9/69	16/366	-	10/69	40/366	-
Time to event, median (IQR), y	2.02 (1.47, 2.79)	2.10 (1.51, 4.10)	-	1.98 (1.44, 2.63)	2.04 (1.40, 4.07)	-
IR, per 100 person-years	5.64	1.53	-	6.54	4.06	-
RD (95% CI)	4.12 (0.36, 7.88)	0 [Reference]	0.032	2.49 (-1.81, 6.78)	0 [Reference]	0.256
sHR (95% CI)	3.72 (1.64, 8.43)	1 [Reference]	0.002	1.53 (0.56, 4.21)	1 [Reference]	0.407
**>1/4 tumor pleural contact**						
No. of events/No. of patients	9/33	15/156	-	5/33	18/156	-
Time to event, median (IQR), y	1.59 (0.63, 2.38)	2.09 (1.51, 3.64)	-	1.84 (1.48, 2.38)	2.33 (1.43, 4.15)	-

IR, per 100 person-years	15.82	3.69	-	9.06	4.30	-
RD (95% CI)	12.12 (1.62, 22.62)	0 [Reference]	0.024	4.75 (-3.11, 12.62)	0 [Reference]	0.236
sHR (95% CI)	4.89 (2.13, 11.22)	1 [Reference]	<0.001	1.97 (0.44, 8.91)	1 [Reference]	0.377
**Pleural/fissural retraction**						
No. of events/No. of patients	8/63	23/341	-	9/63	32/341	-
Time to event, median (IQR), y	2.39 (1.40, 3.76)	2.99 (2.02, 4.61)	-	2.39 (1.41, 3.47)	2.64 (1.96, 4.11)	-
IR, per 100 person-years	4.71	1.97	-	5.55	3.04	-
RD (95% CI)	2.73 (-0.63, 6.09)	0 [Reference]	0.111	2.51 (-1.27, 6.29)	0 [Reference]	0.193
sHR (95% CI)	2.16 (0.98, 4.76)	1 [Reference]	0.057	1.77 (0.61, 5.17)	1 [Reference]	0.295
**Pleural tags with thickening**						
No. of events/No. of patients	6/30	11/126	-	4/30	27/126	-
Time to event, median (IQR), y	2.24 (1.30, 3.38)	2.92 (1.54, 4.85)	-	2.38 (1.46, 3.60)	2.25 (1.30, 3.26)	-
IR, per 100 person-years	8.31	2.68	-	4.71	7.84	-
RD (95% CI)	5.63 (-1.21, 12.47)	0 [Reference]	0.106	-3.13 (-8.78, 2.52)	0 [Reference]	0.277
sHR (95% CI)	2.84 (1.06, 7.63)	1 [Reference]	0.038	0.62 (0.17, 2.22)	1 [Reference]	0.457
**<1/4 contact or non-thickened tags**						
No. of events/No. of patients	1/21	9/269	-	0/21	12/269	-
Time to event, median (IQR), y	2.06 (1.87, 3.37)	2.05 (1.52, 4.04)	-	2.04 (1.92, 3.37)	2.06 (1.52, 3.04)	-
IR, per 100 person-years	1.81	1.21	-	0.18	1.68	-
RD (95% CI)	0.60 (-3.03, 4.23)	0 [Reference]	0.747	-1.50 (-3.00, 0.01)	0 [Reference]	0.052
sHR (95% CI)	1.71 (0.22, 13.33)	1 [Reference]	0.609	0.12 (0.01, 1.32)	1 [Reference]	0.084

Abbreviations: SBRT, stereotactic body radiotherapy; IR, incidence rate; RD, rate difference; sHR, subdistribution hazard ratio; IQR, interquartile range; CI, confidence interval. Median time-to-event period was calculated using the event time for patients experienced events and the last follow-up time for those who did not.

* As no events occurred in the SBRT group after three years, the model was estimated based on data within three years. †The most discriminative cut-off point for solid diameter was determined to be 16.3 using the maximally selected rank statistics methods adapted for time-to-event outcomes with competing risks.

The overlap weights were re-created for each subgroup analysis. If the proportional hazards assumption is violated, a landmark analysis was performed.

Table S4. Mortality and cause of death within 90 days after treatment in SBRT and Surgery Groups

No	Treatment	Sex	Age	Time to Death from treatment (days)	Cause of death	Treatment-related
1	SBRT	M	83	63	Pulmonary thromboembolism and aspiration pneumonia following a femur fracture	No
2	SBRT	M	77	80	Altered mental status from normal pressure hydrocephalus	No
3	Surgery	F	76	71	Postoperative pneumonia	Yes
4	Surgery	M	65	53	Postoperative pneumonia	Yes
5	Surgery	M	78	22	Cardiac arrest	No
6	Surgery	F	76	5	Unknown*	Uncertain
7	Surgery	M	84	62	Aortic dissection complication	No

*After discharge without postoperative complications, death occurred at a hospital outside our facility due to an unknown cause.
